# Emerging Roles of Sodium Glucose Cotransporter 2 (SGLT-2) Inhibitors in Diabetic Cardiovascular Diseases: Focusing on Immunity, Inflammation and Metabolism

**DOI:** 10.3389/fphar.2022.836849

**Published:** 2022-02-28

**Authors:** Lingxiang Xie, Yang Xiao, Shi Tai, Huijie Yang, Shenghua Zhou, Zhiguang Zhou

**Affiliations:** ^1^ Key Laboratory of Diabetes Immunology, Department of Metabolism and Endocrinology, National Clinical Research Center for Metabolic Diseases, Ministry of Education, The Second Xiangya Hospital of Central South University, Changsha, China; ^2^ Department of Cardiovascular Medicine, The Second Xiangya Hospital of Central South University, Changsha, China

**Keywords:** diabetic cardiovascular diseases, inflammation, SGLT-2 inhibitors, immunological factors, metabolism

## Abstract

Diabetes mellitus (DM) is one of the most fast evolving global issues characterized by hyperglycemia. Patients with diabetes are considered to face with higher risks of adverse cardiovascular events. Those are the main cause of mortality and disability in diabetes patients. There are novel antidiabetic agents that selectively suppress sodium-glucose cotransporter-2 (SGLT-2). They work by reducing proximal tubule glucose reabsorption. Although increasing evidence has shown that SGLT-2 inhibitors can contribute to a series of cardiovascular benefits in diabetic patients, including a reduced incidence of major adverse cardiovascular events and protection of extracardiac organs, the potential mechanisms of SGLT2 inhibitors’ cardiovascular protective effects are still not fully elucidated. Given the important role of inflammation and metabolism in diabetic cardiovascular diseases, this review is intended to rationally compile the multifactorial mechanisms of SGLT-2 inhibitors from the point of immunity, inflammation and metabolism, depicting the fundamental cellular and molecular processing of SGLT-2 inhibitors exerting regulating immunity, inflammation and metabolism. Finally, future directions and perspectives to prevent or delay cardiovascular complications in DM by SGLT-2 inhibitors are presented.

## 1 Introduction of SGLT-2 Inhibitors

Diabetes mellitus (DM) is a kind of chronic metabolic diseases. Hyperglycemia is the typical characteristic of DM, which is mainly caused by defective insulin secretion or/and impaired insulin biology. The International Diabetes Federation (IDF) recently released its 10th edition of the Diabetes Atlas. It showed that there are approximately 537 million adults worldwide have diabetes, which means that almost one in ten adults are affected, while almost half are undiagnosed (IDF, 2021). Various acute and chronic complications of diabetes bring serious harm to patients, and their treatment costs are also a heavy burden on society and individuals. Indeed, among people suffering from diabetes, the risk of mortality increased by 4-fold.

The kidney is indispensable for glucose metabolism (Devaraj et al., 2006). During urine formation, blood flows through the kidneys. Glucose in the blood is reabsorbed after glomerular filtration. However, glucose reabsorption is quite different compared with that of water. Glucose does not freely pass through the cell membrane of proximal tubules. It must be transported with the help of glucose transporters from renal proximal tubules named sodium-glucose cotransporters (SGLTs) (Wood and Trayhurn, 2003). Scientists recognized two types of SGLTs: SGLT-1 and SGLT-2 along with further research. SGLT-2 distributed in the S1 segment that plays a dominant role in glucose reabsorption. It is a low-affinity but high-transport transporter. SGLT-2 regulates for about 90% glucose reabsorption, and the remaining is accomplished by SGLT-1 (Turk et al., 1994).

SGLT-2 inhibitors are novel agents working in the proximal renal tubule that selectively block SGLT-2. SGLT-2 inhibitors randomly capture SGLT-2 and block the pathway of glucose reabsorption into the blood circulation, as the affinity between them is much greater than that of glucose, so that the excess glucose is removed in the urine, leading to reduced blood glucose. SGLT-2 inhibitors can also reduce glycosylated proteins, regulate insulin sensitivity and improve beta cell function in liver and peripheral tissues. They can further improve insulin resistance in liver, thereby normalizing higher liver sugar output (Nauck, 2014).

Since the first SGLT-2 inhibitor, dapagliflozin, was approved in 2012 (Vasquez-Rios and Nadkarni, 2020), scientists have developed many SGLT-2 inhibitors on the market, including empagliflozin, canagliflozin, ertugliflozin, ipragliflozin, luseogliflozin and tofogliflozin. In diabetes patients, SGLT-2 inhibitors exhibit effective hypoglycemic activity. To our surprise, they also lower the risk of diabetic cardiovascular disease. Results from landmark EMPA-REG OUTCOME first revealed that in T2DM patients, empagliflozin not only exhibits ability to control glucose effectively, but it also, more importantly, reduces cardiovascular hospitalization and mortality rate (Zinman et al., 2015). More surprisingly, in heart failure patients without T2DM, SGLT-2 inhibitors showed similar clinical benefits. SGLT-2 inhibitors can reduce the occurrence and play a primary prevention role in diabetes patients without previous heart failure. In patients with prior heart failure, they provide secondary prevention by reducing the risk of rehospitalization and cardiovascular death (Cowie and Fisher, 2020).

To focus more specifically on why SGLT-2 inhibitors, but not other antidiabetic drugs, show these cardiovascular benefits in addition to the hypoglycemic effect, the mechanism of diabetic cardiovascular disease from the perspectives of inflammation and metabolism will be first summarized in this review, and the cellular and molecular basis of SGLT-2 inhibitors in regulating cardiovascular effects will be further discussed.

## 2 Role of Inflammation and Metabolism in Diabetic Cardiovascular Diseases

Diabetic cardiovascular diseases broadly describe a class of diabetic complications that affect the heart or vessels. Diabetic cardiovascular diseases, from the clinical manifestations, including coronary atherosclerotic heart disease, diabetic cardiomyopathy and diabetic cardiovascular autonomic neuropathy, the symptoms are similar to some extent, no matter in patients with or without diabetes. Those symptoms include angina pectoris, acute myocardial infarction, heart failure and arrhythmia, but have the following characteristics: 1) The incidence of diabetic coronary heart disease correlates with the severity of diabetes. 2) The incidence of myocardial infarction is high, which may be related to diabetic autonomic neuropathy. 3) The complications of diabetic myocardial infarction, such as cardiac arrest, shock and heart failure, are significantly higher than those of nondiabetic myocardial infarction. Diabetes, obesity and a sedentary lifestyle are considered to be major contributors to disease progression. These risk factors accelerate fats accumulation, cholesterol and inflammatory cells in the coronary arteries, leading to plaque formation and restricted blood flow, a pathology commonly known as atherosclerosis (Swirski and Nahrendorf, 2013; Nahrendorf and Swirski, 2015; Nahrendorf and Swirski, 2017). Ventricular dysfunction is common in diabetic cardiomyopathy. In addition, it may be present in 60% of patients with diabetic hypertension as a pseudonormalization of the diastolic pattern (Lorenzo-Almoros et al., 2017).

Immunometabolism refers to the immune system being intimately linked to other metabolic functions in a way that was never previously recognized. Over the course of evolution, mammals have developed functional systems to communicate the immune system with metabolism. Immunometabolism in the development of diabetic cardiovascular diseases involves the crosstalk of immune cells, including common innate immunocytes and adaptive immunocytes, with cardiac cells like cardiomyocytes, endotheliocytes and extravascular adipose tissue. It has been well established that innate immune cells, especially macrophages, primarily cause the initiation and progression of diabetic cardiovascular diseases. Phagocytosis is the major function of macrophages. It eliminates pathogens to protect hosts. Dead cells are also removed by this way. Macrophages are highly variable cells involved in the preinflammatory and preresolution phases of inflammation (Swirski et al., 2016). Typically, there are two types of macrophages: classically macrophages, known as M1 macrophages, and M2 macrophages (Benoit et al., 2008). Normally, the stability of the cardiac microenvironment is maintained by tissue-resident M2 macrophages, as M2 macrophages exhibit regulatory functions. However, under stress conditions such as diabetes, the homeostasis of the cardiac microenvironment is disrupted, and monocytes in the blood are recruited to the injured cardiac tissue, differentiating into M1 macrophages and secreting a large number of inflammatory factors to promote cardiac tissue damage. Consistent with this, analysis of the macrophage profile revealed that diabetes results in a shift from M2 to M1 macrophages, which show a proinflammatory phenotype (Gallagher et al., 2015). Even more, chemokines recruit monocytes from macrophages. Foam cells are formed by absorbing cholesterol particles, which ultimately promote atheroma development (Libby, 2021). Moreover, excessive cytokines trigger macrophage and smooth muscle cell apoptosis and contribute to necrotic core formation (Back et al., 2019).

Changes in immunometabolism are initiated by cell activation after antigen stimulation. After the stimulus signal is transduced into the cell, the target cell undergoes metabolic reprogramming to support downstream signal activation, leading to changes in cytokine secretion or other function-related events. Cardiovascular endothelial cells express a series of chemokines and adhesion molecules, which attract inflammatory cells, including monocytes and lymphocytes, to endothelial cells. Activated macrophages and lymphocytes can produce many cytokines, and elevated proinflammatory cytokines can induce matrix metalloproteinase secretion (MMP) and MMP inhibitor expression, which can decrease the extracellular matrix synthesis and increase its degradation, aggravating cardiac injury. Consistent with the changes in macrophages, a series of proinflammatory cytokines could be detected in monocytes of DM patients. Compared with control group, IL-1 family, IL-6, and tumor necrosis factor-α (TNF-α) are the major inflammatory factors produced by isolated monocytes (Devaraj et al., 2006; Giulietti et al., 2007).

Therefore, strategies for regulating these immune-metabolic and inflammatory mechanisms are critical steps in the development of potential treatments for diabetic cardiovascular diseases.

## 3 SGLT-2 Inhibitors Improve Diabetic Cardiovascular Diseases by Regulating Immunity

Epidemiological experimental studies have shown that cardiovascular disease has become the most severe diabetic complication (Leng et al., 2016), and SGLT-2 inhibitors exhibit astonishing cardiac protection beyond regulating blood glucose levels. Although the precise mechanism remains unclear, the immunological mechanisms have drawn increasing attention.

### 3.1 The Role of SGLT-2 Inhibitors in Regulating Specific Immune Cells

The precise pathophysiological mechanisms of diabetic cardiovascular disease have not been fully identified. Plenty of evidence shows that chronic inflammatory responses, macrophage infiltration and proliferation are the leading cause (Hansson, 2005; Hansson and Libby, 2006)s.

As we discussed above, atherosclerotic lesions consist of necrotic foam cells, which are formed by CD36 macrophages. During this process, oxidized low-density lipoprotein (ox-LDL) plays a dominant role (Lin et al., 2012). CD36 gene expression from db/db mice and STZ-induced mice was attenuated by SGLT-2 inhibitor dapagliflozin treatment in macrophages, which is associated glucose-lowering effects. Dapagliflozin also exerts anti-atherogenic effects by reducing cholesterol ester accumulation in macrophages to suppress foam cell formation (Terasaki et al., 2015). On the other hand, macrophage infiltration and polarization are pivotal in diabetic cardiovascular disease. Therefore, to control excessive inflammation during the pathogenesis of diabetic cardiovascular disease, restricting macrophage infiltration is critical.

In STZ-induced DM mice, the application of dapagliflozin led to reduced atherosclerotic lesions. This is consistent with the reversed macrophage infiltration. More interestingly, the reduced macrophages within the lesion could in turn stabilize plaques (Leng et al., 2016). Empagliflozin also effectively reduced macrophage infiltration to induce beneficial effects on specific cardiomyopathy (Zhang Q. Q et al., 2020). In regard to macrophage polarization, activating M2 polarization may also exhibit protective effects. In animal models, it has been demonstrated that increased M1 to M2 polarization after SGLT-2 inhibitor empagliflozin or dapagliflozin treatment prevents myocardial injury (Lee et al., 2017; Xu et al., 2019). Isolated macrophage polarization was also directly altered by empagliflozin. Cultured macrophages transform to M2 after empagliflozin is added to the system (Koyani et al., 2020). Apart from activating M2 polarization, SGLT-2 inhibitors could also reduce inflammasome activity to exercise their cardioprotective effect (Xu et al., 2017). More and more evidence showed a causally link of obesity with cardiovascular diseases. Many studies demonstrate that obesity-induced adipose tissue dysfunction contributed to cardiovascular disease to a certain extent. This dysfunction induces chronic inflammatory state within the organism (Fuster et al., 2016). Miyachi (Miyachi et al., 2018) et al. found in a High-Fat-Diet fed mice model, ipragliflozin has the potential to expand healthy adipose tissue, which means that ipragliflozin could induce adipose tissue growth without impair the systemic glucose or lipid metabolism and adipose inflammation, but they do observe a reduced ratio of M1 to M2-like macrophages.

When it comes to the role of adaptive immunity in diabetic cardiovascular disease, the balance between Th17 and Tregs were broken, and an elevated Th17 to Tregs ratio is accompanied with the progress of diabetic cardiovascular disease, methods for promoting Th17 to Tregs transformation are potential treatments for T2DM complications (Wang et al., 2018). Moreover, in diabetes patients, Th17 and Tregs imbalance in connection with high density lipoprotein (HDL) levels in the circulation and following cardiovascular complications (Zeng et al., 2012). However, there are only few studies showed that SGLT-2 inhibitors treatment could change the T cell profile. A 6-months Empagliflozin treatment could improve Tregs protective functions while inhibit Th17. Empagliflozin also exhibit an anti-T cell proliferation effect on CD4^+^ T cells. These effects is believed to improve the cardiovascular complications of the diabetes patients (Borzouei et al., 2021). In addition to the cardiovascular benefits, empagliflozin has the ability to attenuate Th17-mediated inflammatory responses in T2DM with non-alcoholic fatty liver disease (Meng et al., 2021).

### 3.2 The Role of SGLT-2 Inhibitors in Regulating the Immune Signaling Pathway

Macrophages are important in regulating complex inflammatory responses and immunoreactions to induce cardiovascular disease. As we discussed above, the infiltration and polarization of macrophages is influenced by SGLT-2 inhibitors. They could also affect inflammatory cytokine expression through the regulation of the immune signaling pathway.

Several very interesting studies have depicted an interaction between SGLT-2 inhibitors and the NF-κB (nuclear factor-kappa B) signaling pathway. Onofrio (D'Onofrio et al., 2021) et al. found that in T2DM patients, SGLT-2 inhibitors canagliflozin pretreatment could effectively downregulate SLC5A2 gene expression. The attenuated SGLT2 expression is related with reduced expression of NF-κB on human aorta endothelial cells. Empagliflozin reduces the activity of mTOR, upregulates the nuclear factor erythroid 2-related factor/heme oxygenase-1 (Nrf2/HO-1) pathway to control and limit oxidative stress overactivation, and downregulates inflammatory responses by controlling the NF-κB pathway on isolated cardiomyocytes (Sun et al., 2020). Another study demonstrated that SGLT-2 inhibitors regulate the macrophage Toll-like receptor 4/NF-κB signaling pathway to regulate inflammation (Lee et al., 2020).

Moreover, the JAK/STAT signaling pathway has a wide range of functions in modulating immune responses (Ihle and Kerr, 1995). STATs are a distinct family of proteins. Previous studies have shown that there is a strong correlation between STAT activation and cardioprotective mechanisms. When pre-treat macrophages were with LPS, empagliflozin application reduced the systemic inflammatory response through the downregulation of macrophage JAK2/STAT1 signaling pathways. Besides, the macrophage IKK/NF-κB and MKK7/JNK pathways were also inhibited. This leads to the reduction of cyclooxygenase-2 (COX-2) and prostaglandin E2 (PGE2) expression (Lee et al., 2021). The reperfusion injury signaling kinase (RISK) pathway was proven to be associated with cardioprotective effects (Schulman et al., 2002). Empagliflozin improves cardiac cell survival through ERK1/2 pathways. Activated RISK pathway in the ventricles leads to a series of follow-up effects, which contribute to cardioprotection from ventricular arrhythmias (Hu et al., 2021).

Apart from the classical immune signaling pathway, few studies have been conducted to investigate the interaction between SGLT-2 inhibitors and miRNAs in pathogenesis of diabetic cardiovascular disease. Zhang (Zhang W. Y et al., 2020) et al. found a significantly increased miRNA-30d expression in diabetic cardiomyopathy rats. Antagonistic miR-30D can significantly improve cardiac function and prevent myocardial fibrosis in diabetic animals. The same protective effect was obtained with SGLT-2 inhibitors. SGLT-2 inhibitors promote cardiac autophagy, improve cardiac function by inhibiting the miRNA-30d/KLF9/VEGFA pathway.

## 4 SGLT-2 Inhibitors Improve Diabetic Cardiovascular Diseases by Regulating Inflammation

### 4.1 The Role of SGLT-2 Inhibitors in Regulating Inflammatory Factors

Macrophages are the main cell component in diabetic cardiovascular disease, and their secretion of a variety of cytokines can change the local environment, causing the development of the disease. As we mentioned before, the development of cardiovascular disease relies largely on a variety of immunological factors (Kang et al., 2020). There is much evidence showing that the major inflammatory cytokines during this process are the IL-1 family (Carty et al., 2009; Herder et al., 2017; Pfeiler et al., 2019), IL-6 (Carty et al., 2009; Prochnau et al., 2012; Morieri et al., 2017), IL-17 (Madsen et al., 2016), IL-18 (Buraczynska et al., 2016; Lee et al., 2019), IL-32 (Damen et al., 2017), TNF-α (Singh et al., 2005; Shen et al., 2016), C-reactive protein (CRP) (Chiang et al., 2019; Martin-Nunez et al., 2020). Adhesion Molecules are also very important, such as vascular cell adhesion molecule-1 (VCAM-1) (Denys et al., 2016; Kunutsor et al., 2017; Cao et al., 2020; Yari et al., 2020), intercellular adhesion molecule-1 (ICAM-1) (Cao et al., 2020; Yari et al., 2020), and platelet endothelial cell adhesion molecule-1 (PECAM-1) (Stevens et al., 2008). SGLT-2 inhibitors are recognized as effective agents for decreasing these inflammatory factors.

#### 4.1.1 Cytokines and Chemokines

Empagliflozin treatment exhibits systemic anti-inflammatory effects by reducing IL-1β secretion. Whether empagliflozin was applied in atherosclerosis model or cocultured with macrophages, it significantly decreased the production of the IL-1β (Liu et al., 2021). Aside from the reduction in IL-1β, another study showed that the application of canagliflozin restricted the secretion of IL-6, MCP-1 and reduced the mRNA levels of these cytokines. These cytokines are stimulated by IL-1β and activated via the AMPK pathway. What’s more interesting, in diabetes patients, canagliflozin treatment also displayed an anti-arteriosclerotic effect on blood vessels. This effect is independent of the hypoglycemic effect (Mancini et al., 2018). Another interesting study revealed that canagliflozin treatment reduced the production of IL-6 by endothelial cells (Uthman et al., 2021). When endothelial cells were pretreated with high glucose medium *in vitro*, canagliflozin attenuated the production of IL-6. In addition, the secretion of other cytokines like IL-18, TNF-α was also impaired systemically (Winiarska et al., 2021). When pre-treat mouse with LPS, canagliflozin also significantly decreased both TNF-α and IL-6 levels in the circulation (Xu et al., 2018). TNF-α is a key factor during the progression of cardiac fibrosis. It initiates the inflammatory response and leads to cardiac cell injury (Duerrschmid et al., 2013). Empagliflozin cocultured with cardiomyocytes also plays a protective role by reducing TNF-α secretion. Moreover, the mRNA level of TNF-α is also impaired. This is consistent with inducible nitric oxide synthase expression. These factors work together to maintain the energy balance of cardiac cells. Empagliflozin treatment also significantly reduced IL-18 and IL-1β levels to prevent diastolic dysfunction onset and development (Byrne et al., 2020).

#### 4.1.2 Adhesion Molecules

During the process of inflammatory cell infiltration in cardiac tissue, it has been clearly demonstrated in many studies of both patients and experimental animal models that adhesion molecule expression plays a key role (Franssen et al., 2016).

Empagliflozin treatment in obese rats and isolated HFpEF human cardiomyocytes could significantly decrease adhesion molecule levels, especially ICAM-1 and VCAM-1 (Kolijn et al., 2021) levels. Moreover, high glucose increased coronary artery cultured endothelial cells express VCAM-1, while empagliflozin is highly effective in preventing high glucose-induced vascular endothelial dysfunction (Khemais-Benkhiat et al., 2020). Another study also showed a similar result that empagliflozin treatment is beneficial for improving endothelial dysfunction in obese rat model by reducing VCAM-1 (Park et al., 2020). In addition, empagliflozin is thought to reverse cardiac remodeling to a certain degree. These results suggest SGLT-2 inhibitors have protective properties on endothelial cells and vascular systems. Canagliflozin could also prevent endothelial dysfunction in some studies. In diabetic ApoE KO mice, 12 weeks of canagliflozin treatment decreased atherosclerotic lesions, while 8 weeks of treatment significantly decreased endothelial dysfunction. When analyzing the adhesion molecule profiles in the aorta, all treated mice showed significantly impaired ICAM-1 and VCAM-1 production (Rahadian et al., 2020). Another study demonstrated that ipragliflozin also improved hyperglycemia-induced endothelial dysfunction following the similar mechanisms described above. In the abdominal aorta, VCAM-1 and ICAM-1 levels are significantly reduced (Salim et al., 2016). PECAM-1 is another important adhesion molecule in diabetic cardiovascular disease progression. The dominant function is to mediate immunocyte trafficking. Studies have shown a correlation of PECAM-1 with vascular integrity (Wimmer et al., 2019). One study showed that the application of luseogliflozin in a short period of time is sufficient to downregulate PECAM-1 and ICAM-1 gene expression in STZ-treated ApoE KO mice (Nakatsu et al., 2017).

### 4.2 The Role of Anti-inflammatory Agents on SGLT-2 Expression

SGLT-2 express in both the kidney and small intestine. Unlike SGLT-1, SGLT-2 expression in cardiac and vascular tissues has not been reported yet (Alshnbari et al., 2020). It is interesting to remark that agents with anti-inflammatory function could also target SGLT-2 and those agents inhibit SGLT-2 expression. Sardu (Sardu et al., 2021a) et al. first found that SGLT2 expression in the pericoronary fat is increased during acute myocardial infarction progressing, which is accompanied by over-inflammation. Interestingly, after metformin treatment, excessive inflammation is controlled, SGLT2 level is also reduced, this may partially explain why metformin therapy might ameliorate cardiovascular outcomes. In addition to diabetic cardiovascular disease, there is another study demonstrated that in heart failure rat model, tonic renal sympathetic nerve showed increased activation, which enhances renal SGLT-2 expression and its functional activity (Katsurada et al., 2021). Interestingly, Marein, agent with anti-inflammatory properties, could improve diabetic nephropathy in db/db mice. It ameliorated metabolic dysfunction in high glucose-treated HK-2 cells by inhibiting renal SGLT-2 (Guo et al., 2020).

### 4.3 The Anti-inflammatory Effects of SGLT-2 Inhibitors Lead to the Amelioration of Clinical Outcomes

The exact mechanisms between diabetic cardiovascular disease and inflammation are complex and remain unclear (Sardu et al., 2020). As we described above, SGLT-2 inhibitors are novel agents in controlling hyperglycemia, their cardioprotective properties may result from the effect of reduced glucose level, we define this as glucose dependent effects. On the other hand, during the process of diabetic cardiovascular disease, inflammatory factors can be activated and secreted in the site of inflammation, it can be released into the circulation and detected as indicators of inflammatory status. SGLT-2 inhibitors regulate those inflammatory factors and exhibit anti-inflammatory effect.

#### 4.3.1 Glucose Dependent Effects

Canagliflozin and dapagliflozin have been confirmed to ameliorate glycemic parameters, when use alone or in combination (Meng et al., 2021). A 52-week comparison between sitagliptin and different dose of canagliflozin demonstrated that high dose canagliflozin showed improved glycaemia and bodyweight, as well as reduced glycated hemoglobin (HbA1c) (Lavalle-Gonzalez et al., 2013). Another study showed that 52-week dapagliflozin treatment improved HbA1c level and reduced body weight, despite glycemic efficacy between dapagliflozin and glipizide (Ku et al., 2019).

#### 4.3.2 Glucose Independent Effects

Regards the glucose independent effects of SGLT-2 inhibitors. There is a research group found that SGLT-2 inhibitor could significantly attenuate circulation inflammatory burden and improve clinical outcomes (Sardu et al., 2021b). Another study showed that in non-infarcted myocardium of rats, empagliflozin could attenuate inflammatory, modify cardiac energy metabolism, and reduce oxidative stress. These effect are conducive to the prevention of diabetes induced post myocardial infarction mortality (Oshima et al., 2019). Lim (Lim et al., 2019) et al. get similar result that canagliflozin attenuates myocardial infarction through intermediate signaling mechanism, which is a glucose-independent cardiac survival pathway.

## 5 SGLT-2 Inhibitors Improve Diabetic Cardiovascular Diseases by Regulating Cardiac Metabolism

The heart has a constant need for energy and consume a variety of substrates like free fatty acids and glucose. Under stress conditions such as T2DM and heart failure (HF), impaired glucose utilization leaves the heart without adequate energy sources. Under this extreme occasion, fatty acids and ketones provide an alternative energy source. The metabolism of these secondary energy stocks provide more ATP for the heart (Sowton et al., 2019; Tran and Wang, 2019; Zelniker and Braunwald, 2020).

In patients with T2DM, systemic and myocardial insulin-mediated glucose utilization is impaired. Free fatty acids (FFAs) is more reliable to produce sufficient energy. However, the oxygen consumption for metabolizing FFAs is greater than that of glucose (Correale et al., 2021). This metabolic state leads to reduced cardiac metabolic efficiency and inadequate ATP production. Several studies have shown that in diabetes patients, SGLT-2 inhibitor treatment exhibits protective effects by improving cardiac energy metabolism. Empagliflozin promotes ketone *β*-hydroxybutyrate (*β*OHB). Some studies indicate that βOHB may be a cheaper source of energy to act as a “superfuel” in patients with diabetic cardiovascular disease (Ferrannini et al., 2016).

As we mentioned above, glucose is no longer the primary source of energy during diabetes and HF. Empagliflozin improves myocardial energy metabolism and systolic function and reverses cardiac remodeling by stimulating the conversion of energy sources to ketone bodies, thus improving poor cardiac remodeling in HF patients (Gaborit et al., 2021). In a fasting diabetic db/db mouse model, empagliflozin treatment reduced cardiac preload and increased plasma ketones. Elevated ketone levels are positively correlated with cardiac ATP production (Abdurrachim et al., 2018). Moreover, ketone bodies are not just recognized as an alternative source of energy. They can also inhibit nucleotide-binding oligomerization domain-like receptor P3 (*NLRP3*) pathways to act as an inflammation suppression role (Youm et al., 2015; Zelniker and Braunwald, 2020). It is interesting to note that another study disagrees with this. This group demonstrated that when mice were treated with empagliflozin, heart failure symptoms improved. However, 31% more ATP production was not due to ketones. They only observed enhanced oxidation of traditional energy sources, including glucose and fatty acids (Verma et al., 2018).

## 6 Conclusions and Perspectives

**FIGURE 1:**
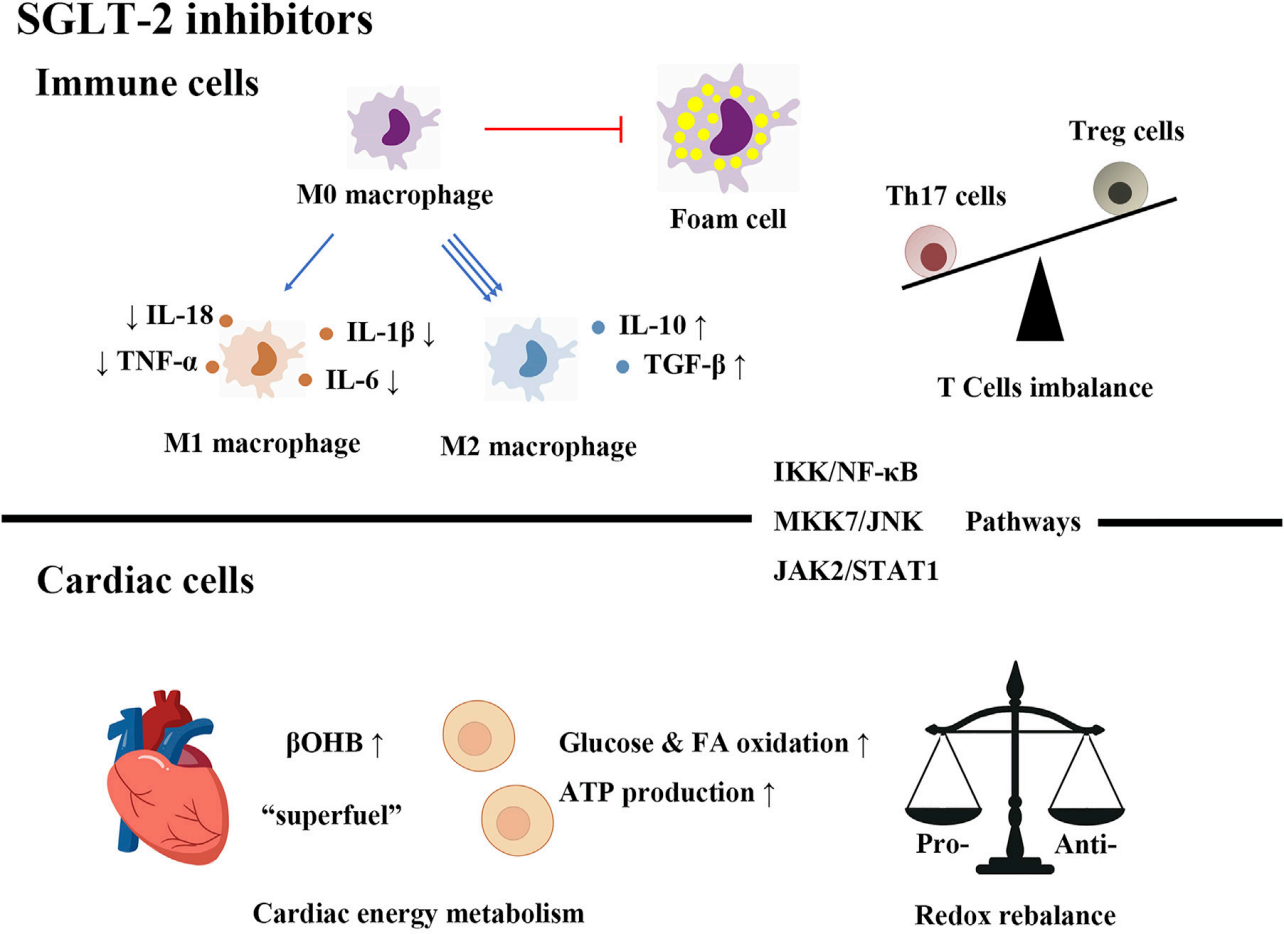
SGLT-2 inhibitors improve diabetic cardiovascular diseases by regulating immunity, inflammation and metabolism.

SGLT-2 inhibitors are novel agents for controlling hyperglycemia. Their action does not depend on the secretion of insulin. They are highly effective and safe hypoglycemic drugs in the treatment of T2DM as well as cardiovascular complications, which can be used in daily clinical practice, facilitating further research on different cardiovascular diseases. In this review, we summarize the role of immunity, inflammation and metabolism in diabetic cardiovascular diseases, highlight the protective effects of SGLT-2 inhibitors on the heart, list the possible cardioprotective mechanisms of SGLT-2 inhibitors from an immunological, inflammatory and metabolic perspective (Figure 1). The exact cardiovascular beneficial mechanisms of SGLT-2 inhibitors have not been fully elucidated, and short-term benefits and long-term benefit mechanisms may be different. Cardiac protection of SGLT-2 inhibitors can be mediated by the following mechanisms: 1) regulating immune cell infiltration and polarization, promoting an anti-inflammatory immune cell profile and inhibiting foam cell formation. 2) Restricting the expression of proinflammatory factors and inducing anti-inflammatory cytokine production through the activation of multiple signaling pathways. 3) It prevents oxidative stress and regulates cardiac metabolism, which provides alternative cardiac energy sources and reduces oxidative injury.

There might be some interactions between different mechanisms. Let us suppose that the cardioprotective effects come from a combination of systemic mechanisms. The crosstalk between immunocytes and cardiac cells is not a single fact that plays a huge role. On the other hand, whether the mechanism found in animal experiments is also suitable for humans remains to be further verified, and more relevant studies are needed in the future to explain the above doubts.

Although SGLT-2 inhibitors have many advantages, they still have the following problems: urinary tract infection, reproductive tract infection, diabetic ketoacidosis, etc. However, these risks can be prevented if used with caution. It is still hoped that people pay more attention to their clinical use. Deeper research is needed to reveal the exact mechanisms of SGLT2 inhibitors under its cardiac benefits.
